# Biochar and zinc oxide nanoparticles partnership: a multi-faceted strategy to enhance rice productivity and remediate antimony polluted soils

**DOI:** 10.1186/s12870-026-09069-6

**Published:** 2026-06-12

**Authors:** Haiying Tang, Wenjie Jiang, Yulong Ma, Dongguang Wang

**Affiliations:** School of Agriculture and Biotechnology, Hunan University of Humanities, Science and Technology, Loudi, 417000 China

**Keywords:** Antimony, Biochar, Gene expression, Nano-particles, Nutrient uptake

## Abstract

**Background:**

Antimony (Sb) is a toxic metalloid that poses a significant threat to living organisms and ecosystem sustainability. Biochar (BC) and zinc oxide nano-particles (ZnO-NPs) are promising amendments to mitigate heavy metals toxicity. However, the potential role of BC and ZnO-NPs in alleviating the Sb toxicity remains unexamined. To address this gap, the present study investigated how BC and ZnO-NPs mitigate Sb toxicity in rice.

**Methodology:**

The study included eight different treatments: control, biochar (2%), ZnO-NPs (100 mg L^− 1^), BC + ZnO-NPs, Sb stress (250 mg kg^− 1^), Sb stress + BC (2%), Sb stress + ZnO-NPs, and Sb stress + BC (2%) + ZnO-NPs.

**Results:**

Antimony suppressed rice growth and yield by inducing oxidative stress (increased hydrogen peroxide (H_2_O_2_) and electrolyte leakage), enhancing soil Sb availability and its accumulation in plant tissues, while reducing hormones synthesis and soil nutrients availability. Biochar + ZnO-NPs markedly enhanced the rice yield by decreasing H_2_O_2_ synthesis (26.06%), EL production (25.68%), soil Sb availability (41.80%) and increasing antioxidants activities, formation of iron plaque in roots (63.60%) and soil nutrients availability. Additionally, BC + ZnO-NPs upregulated the antioxidant genes (*OsAPx6*, *OsCAT*, *OsPOD*, and *OsSOD*), and downregulated the expression of *OsSMP* and *OsMTP1* genes associated with Sb transport, thus helping to counteract Sb toxicity.

**Conclusion:**

These findings suggest that the BC + ZnO-NPs can effectively mitigate the Sb toxicity and improve rice productivity. These findings provide insights to develop the measure for remediating Sb-polluted soils, while simultaneously increasing the safe and sustainable crop production.

**Supplementary Information:**

The online version contains supplementary material available at 10.1186/s12870-026-09069-6.

## Introduction

Antimony (Sb) is a toxic metalloid posing serious ecological and health risks [1]. The recent industrialization, mining, and urbanization have significantly increased its concentration in the environment, which is a serious challenge for living organisms [2]. Antimony is a non-essential metal for humans; therefore, its intake causes skin problems, heart and liver diseases, kidney damage, and cancer [3]. Antimony is widely used in electronic materials, smelting, and ammunition, and its concentration in some areas reached very high toxic levels [4, 5]. For instance, in the Xikuangshan mine of Hunan province, the soil and water Sb have been recorded at 1565 mg kg^− 1^ and 47.4 mg L^− 1^, exceeding the allowable limits [6]. Antimony is easily absorbed by plants due to its presence in two distinct forms: Sb(III) and Sb(V) [7]. Antimony adversely impacts soil nutrient availability, microbial and enzyme activities, and soil structure, consequently reducing plant growth [4, 7, 8]. It is non-essential for plants and impairs electron transport, redox balance [9], and causes oxidation of cellular structures, thereby decreasing growth and yield [10]. Besides this, it also decreases seed germination, seedling growth, water uptake [4], efficiency of PS-II, and microbial activity, ultimately causing growth and yield losses [11, 12]. Plants use different strategies, including antioxidants, osmolyte synthesis, and metal chelation in vacuoles to counteract phyto-toxicity of toxic metals [13]. Nevertheless, toxic metals compromise antioxidant activities [14], therefore, exogenous amendments must be applied to counter their toxicity. Different strategies such as biochar (BC), sorbents, polymers, nano-particles (NPs), and hormones are being used globally to counteract Sb toxicity [15]. Among these strategies, BC, being a carbon-rich material, possesses excellent ability to mitigate Sb toxicity thanks to its alkaline nature with porous surface and functional groups [16–19]. Its field application improves soil structure, carbon, and water availability and changes Sb to a more stable form, thereby ensuring better plant growth [20]. Moreover, BC also improves antioxidant activity, osmo-protectant synthesis, photosynthetic efficiency, and reduces the Sb accretion in plant organs, thereby ensuring better plant performance [11]. Additionally, BC causes Sb immobilization, favors nutrient uptake and microbial activities involved in metals degradation and nutrients, thereby ensuring better growth [8, 20, 21].

Nanotechnology has shown tremendous potential to enhance crop productivity and mitigate heavy metals toxicity [22]. Nano-particles enter the plant tissues and improve plant physiological functioning, metabolic processes, and mitigate heavy metals toxicity [23, 24]. They also improve photosynthetic efficiency, decrease oxidative damage, and metal accumulation [25, 26]. Furthermore, Hussan et al. [27] witnessed that ZnO-NPs mitigated the cadmium toxicity in alfalfa by protecting the plants from ultra-structural damages and increasing antioxidants, and decreasing the cadmium uptake and accumulation. Rice is a staple food for half of the world’s population; nevertheless, it is susceptible to Sb accumulation due to its anaerobic growth in flooded conditions [28]. Rice is the primary source of Sb entry into humans, thereby posing significant health issues [29]. Thus, remediating Sb-polluted soils is a great challenge, and it needs the development of promising measures to counter Sb toxicity and ensure the safe and sustainable crop productivity. Biochar has been extensively studied for mitigating Sb toxicity; however, the role of nanoparticles—particularly ZnO-NPs—in mitigating Sb stress is poorly understood, with only limited evidence available [30]. Moreover, no research has yet examined the combined application of BC + ZnO-NPs, leaving their potential synergistic effects unexplored. The mechanistic insights into how these amendments regulate gene expression, hormonal balance, and redox homeostasis under Sb stress are also lacking. Importantly, rice-specific studies are scarce despite its high vulnerability to Sb accumulation and its significance as a staple food. Thus, we hypothesized that combined BC + ZnO-NPs would alleviate the Sb toxicity in rice through a dual action mechanism: BC would immobilizes soil Sb, while ZnO-NPs would promote iron plaque formation on roots, restricting Sb uptake. Concurrently, they would upregulate antioxidant defense to reduce oxidative stress, suppress expression of Sb-transport genes (*OsSMP* and *OsMTP1*), and enhance soil nutrient availability, thereby restoring hormonal balance and improving growth and yield. The study has following objectives; to explore the effects BC and ZnO-NPs on rice yield, nutrient homeostasis and plant physiological functioning, ii) to explore the role of BC and ZnO-NPs in regulating antioxidant activities, hormonal balance, and gene expression, iii) to study role of BC and ZnO-NPs in Sb partitioning to plant organs, soil nutrient retention and soil Sb availability. These findings provide insight into preventing the Sb accumulation of Sb in rice while ensuring the safe and sustainable crop productivity.

## Materials and methods

### Experimental site

This study was carried out in an open-air wire house equipped with a rain-protected shelter at Hunan University of Humanities, Science and Technology. Soil was obtained from the surface layer (0–20 cm) of the experimental field to perform the study. The collected soil was then sieved (2 mm) to remove debris, and a homogenized sample was taken to analyze various soil properties. The soil was highly acidic (5.55 pH) with a total nitrogen (TN) concentration of 1.74 g kg^-1^, and available phosphorus (AP) and available potassium (AK) concentrations of 26.22 and 119.56 mg kg^-1^, respectively. The soil was treated with C_8_H_4_K_2_O_12_Sb_2_ to obtain a 250 mg kg^-1^ Sb concentration. Thereafter, plastic pots (30 × 24 × 15 cm) were filled with soil and placed in dark conditions for two months by maintaining 70% field capacity. Following this, water was added to the pots, and five seedlings (25 days old) were planted in every pot. Weiliangyou-8612 was used as a test variety and its seed was purchased from local agriculture market. The pots were kept with a 2–3 cm water level, while other practices were uniformly performed to ensure good growth. The data on different traits were collected by using standard methods, and analytical grade chemicals with sterilized instruments were used for different analysis.

### Experimental treatments

**Fig. 1:**
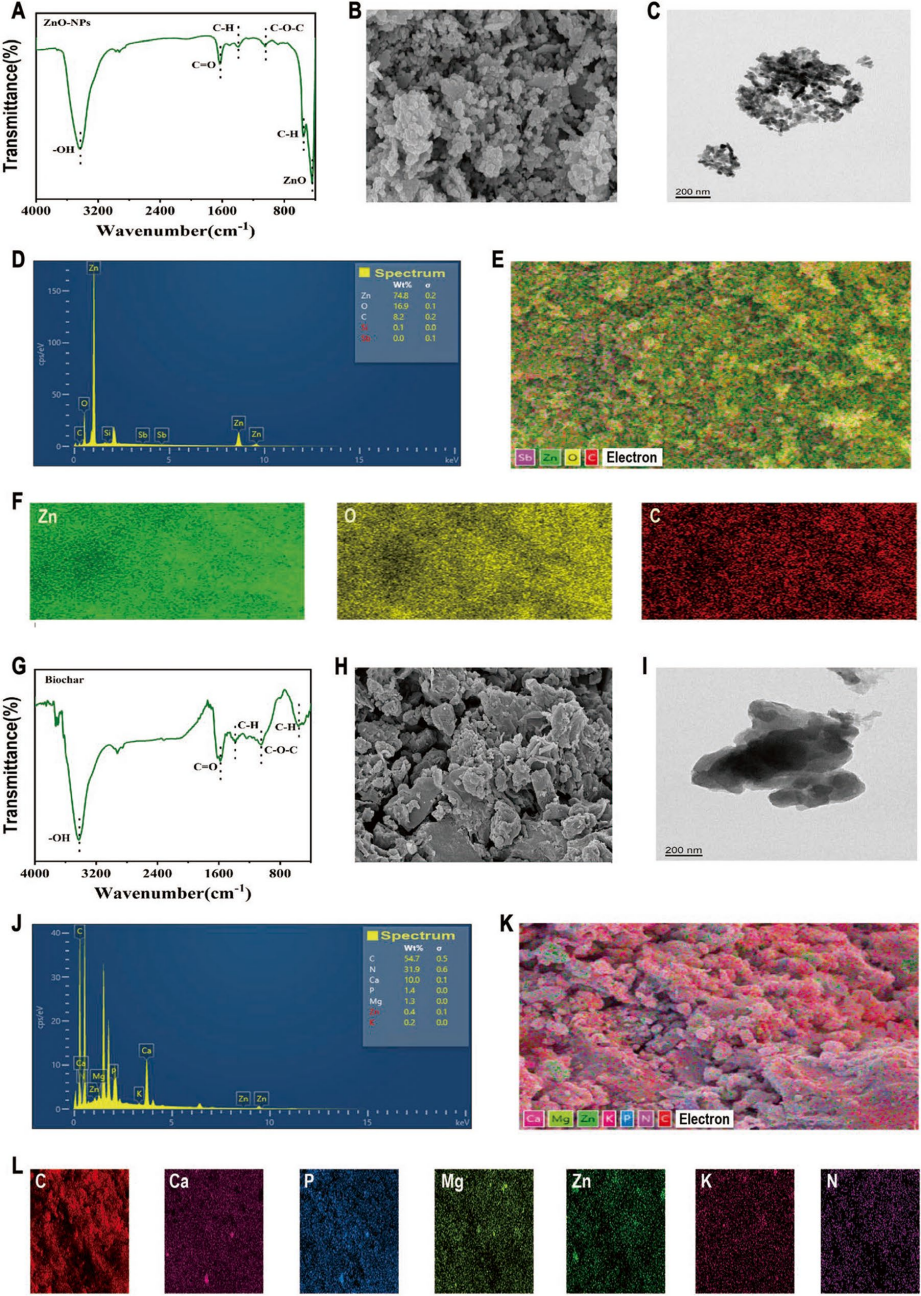
Fourier transform infrared (FTIR: **A**), SEM (**B**), TEM (**C**) and EDS (**D**-**F**) analysis of ZnO-NPs. FTIR (**G**), SEM (**H**), TEM (**I**), and ESD (**J**-**L**) analysis of biochar

The study had eight treatments: control, biochar (2%), ZnO-NPs (100 mg L^-1^), BC + ZnO-NPs (100 mg L^-1^), Sb stress (250 mg kg^-1^), Sb stress + BC (2%), Sb stress + ZnO-NPs (100 mg L^-1^), and Sb stress + BC (2%) + ZnO-NPs (100 mg L^-1^). The experiment followed a complete randomized design with three biological replications. The concentration of Sb was chosen from Hu et al. [11] documenting that 250 mg kg^-1^ caused a significant decrease in rice growth. This concentration was selected from our previous study indicating that this concentration causes measurable physiological and phytotoxic impacts without completely diminishing plant growth. Biochar rate (2%) was selected from earlier studies reporting that 2% BC significantly decreased the Sb toxicity [31]. Furthermore, the rate of ZnO-NPs was selected from previous studies documenting ZnO-NPs (50–100 mg L^-1^) effectively mitigates metals toxicity [32]. ZnO-NPs had a size of 20 nm and they showed a zeta-potential of 26.1 mV in water indicating a good electrostatic stabilization. The biochar used in the study was produced by pyrolysis (500 °C) of rice straw, following a heating rate of 5 °C min^-1^ under limited oxygen supply. Biochar had 9.87 pH with a CEC of 11.22 cmol kg^− 1^. Both BC and nano-particles were subjected to scanning electron microscope (SEM), transmission electron microscope (TEM), energy dispersive X-ray Spectroscopy (EDS) and Fourier transform infrared (FTIR) analysis and details are given in results section. An adequate solution was prepared by mixing the ZnO-NPs in water. Furthermore, surfactant (Tween 20) was added to the solution at a rate of 0.05% to ensure the retention of ZnO-NPs on the leaf surface. This lower concentration of Tween-20 is considered inert and does not affect plant physiological functioning. The sole purpose of Tween-20 addition was to improve the adhesion of droplets. Foliar spray of ZnO-NPs was done for five successive days after 15 days of transplanting. Furthermore, information on the surface morphology and properties of BC is presented in Fig. 1.

### Measurement of photosynthetic pigments, oxidative markers, osmolytes and hormones

To measure the photosynthetic pigments, fresh leaves of rice (0.5 g) were ground in 80% methanol. Following centrifugation, the absorbance of the mixture was measured at 665, 649, and 470 nm to quantify chlorophyll a, chlorophyll b, and carotenoid content [33]. The leaf relative water contents (RWC) were measured with this equation: (FW − DW)/(TW − DW)×100 as suggested by Chattha et al. [34]. The electrolyte leakage was also measured based on measuring electrical conductivity twice by following the methods of Chattha et al. [34]. The concentration of anthocyanin was determined by grinding 0.5 g of leaves in a potassium phosphate buffer (PPB) after reading absorbance at 600 nm [35]. To determine malondialdehyde (MDA) and hydrogen peroxide (H_2_O_2_) concentrations, 0.5 g of leaves was ground in 5 mL trichloroacetic acid (TCA: 0.1%) solution. To determine MDA levels, the mixture was centrifuged at 12,000 rpm. Then, 5 mL of thiobarbituric acid was added, and the mixture was heated at 95 °C for 30 min. After cooling, MDA content was estimated by measuring absorbance at 532 nm and 600 nm [36]. For H_2_O_2_, the leaf extract was collected and added with 100 µL potassium phosphate and 100 µL potassium iodide. Thereafter, it was heated in a water bath (95 °C) and absorbance was read at 390 nm [37]. To estimate the leaf free amino acids, leaf samples were homogenized in 5 mL PPB, and the extract was collected. Then, we mixed the extract with ninhydrin and pyridine and heated it (90 °C) for 30 min, and later, the absorbance was measured at 570 nm. Moreover, total soluble protein was measured by mixing the 100 µL extract and 2 mL Bradford solution after taking absorbance at 595 nm [38]. To estimate the leaf proline concentration, fresh rice leaves (0.5 g) were ground using 10 mL of sulfosalicylic acid solution. Later, it was mixed with ninhydrin and heated (90 °C) for two hours, and the proline concentration estimate after reading absorbance at 520 nm [39]. The synthesis of gibberellin (GA), auxin (IAA), abscisic acid (ABA) and jasmonic acid (JA) was estimated with kits obtained from Comin Biotechnology Co., Ltd., Suzhou, China.

### Measurement of antioxidants activities

The fresh rice leaves were collected and homogenized using PPB. Thereafter, the extract was collected and subjected to centrifugation (8,000 rpm), and the supernatant was used to measure antioxidant activity. To estimate ascorbate peroxidase (APX) activity, 0.5 mM of H_2_O_2_ and 100 µL of enzyme were mixed, and APX concentration was assessed after taking absorbance at 290 nm [40]. For measuring the catalase (CAT), the standard methods of Aebi [41] were used. Briefly, enzyme extract and H_2_O_2_ were mixed, and CAT activity was measured after H_2_O_2_ reached a disappearing point [41]. Moreover, to estimate peroxidase (POD), a mixture constituting the 100 µL enzyme extract, 300 mL H_2_O_2_, and 50 mM PPB was prepared. Thereafter, absorbance reading was done at 470 nm to estimate POD concentration as suggested by Chanes and Mahely [42] (1996). In the case of superoxide dismutase (SOD); the reaction mixture containing 100 enzyme extracts along with 50 µL riboflavin, 25 µL PPB, 100 µL Triton, and 400 µL H_2_O_2_ was made and absorbance reading was done at 560 nm [43].

### Gene expression analysis and root iron plaque formation

Fresh rice leaf samples were collected and used to measure the expression level of different genes. Briefly, 100 mg leaves were used to extract the RNA by using the RNA extraction kit MiniBEST; Takara, China. Then RNA concentration and quality were measured with a NanoDrop spectrophotometer. Later, the extracted RNA was converted to cDNA by using the transcription kit (HiScript^®^ III RT SuperMix). Furthermore, qRT–PCR analysis was carried out using the SuperReal PreMix Plus (SYBR Green) kit, and gene expression level was measured by the procedures of Livak and Schmittgen [44]. Moreover, *OsActin* was used as a reference gene, while information about primers is given in Table S1. The rice roots were taken, and the concentration of iron plaque was measured by the ascorbic citrate acetic (ACA) extraction technique. Briefly, to prepare the mixture of ACA, 5 mL sodium acetate (10%), 40 mL sodium citrate (0.3 M), and 3 g ascorbic acid were mixed. Then, the root samples (1 g) were added to the mixture and subjected to stirring for 3 h. Following this, the mixture was filtered into 100 mL glass flasks, and the root samples were washed three times. The solution was transferred to flasks, adjusted to 100 mL, and Sb and Fe concentrations were measured using atomic absorption spectrometry (AAS).

### Measurement of agronomic traits, tissue nutrients and antimony concentration and soil properties

Plant height was measured from five randomly selected plants, likewise length of five panicles were measured. A sub-sample of grains was taken and weighed to determine the 1000-grain weight (TGW). The pots were harvested to measure biomass yield (BY) and grain (GY). To determine the tissue’s nutrient concentration, plant samples were digested by using HNO_3_ and HClO_4_ in a ratio of 2:1. Then, the volume was made to 50 mL, which was used for subsequent nutrient determination. Nitrogen concentration was measured with the Kjeldahl method, P concentrations were determined with a spectrophotometer, while Ca, K, and Mg concentrations were measured with a flame photometer. The plant samples were dried and ground into powder. Then they were digested by placing them in tubes containing 2 mL HNO_3_. The digestion process was carried out for 2 h at 150 °C. Thereafter, samples were heated (90 °C) to get clear solution and later Sb concentration was measured with AAS. The soil pH was measured with calibrated pH meter in soil and water solution (1:5). The soil nitrogen, phosphorus and potassium was measured with Kjeldahl, Olsen, and ammonium acetate methods, while enzymes activity was estimated with procedures of Tu et al. [45]. Furthermore, to assess soil Sb concentration, soil samples (500 mg) were digested by using HNO_3_ and HCl in a 1:3 ratio. After cooling down, the volume increased to 50 mL, and the concentration of Sb was measured with an atomic absorption spectrophotometer.

### Statistical analysis

The collected data were subjected to normality testing (Kolmogorov-Smirnov test) and homogeneity of variances testing (Bartlett’s test) prior to analysis. Thereafter, data meeting these assumptions were analyzed by ANOVA using Statistix 8.1. Mean differences were evaluated using Tukey’s honestly significant difference (HSD) test at *P* < 0.05. All graphical illustrations in this paper were produced with Sigmaplot-10.

## Results

### Characteristics of biochar and nano-particles

The SEM and TEM analysis of ZnO-NPs confirmed that they have a spherical shape of particle shape. Furthermore, EDS analysis of ZnO-NPs showed that ZnO-NPs contained 8.19% carbon, 16.87% oxygen, 0.11% silicon, and 74.83% zinc (Fig. 1). The peaks of FTIR analysis of ZnO-NPs showed presence of alcoholic compounds, C = C in alkene groups, stretching of C-N in amine groups, C = C in alkene groups and stretching of S = O groups in ZnO-NPs respectively (Fig. 1). The results of SEM and TEM analysis showed that BC had a porous structure, indicating its potential to absorb toxic metals. The FTIR analysis showed that BC contained different functional groups that have the potential to adsorb toxic metals. The peaks of FTIR analysis were observed at 3434.31, 1628.27, 1445.71, 1035.08, and 523.18 cm^− 1^, showing that BC contained a significant amount of carbonyl and carboxylic groups. The peaks observed at aforementioned values indicate that BC contained O-H, C = O, C = C, C-O, and C-H groups [46, 47]. Biochar had 54.72% carbon, 31.89% N, 1.39% phosphorus, 10% calcium, 1.34% magnesium, 0.25% potassium, and 0.42% zinc.

### Growth and yield attributes

Antimony diminished rice growth and morphological traits (Table 1). Antimony significantly decreased the root length (RL: 59.89%), roots fresh weight (RFW: 43.73%), and roots dry weight (40.26%) (Table 1). Notably, co-applied BC + ZnO-NPs significantly enhanced RL (37.12%), RFW (46.86%) and RDW (32.25) in Sb-polluted soil (Table 1). Antimony stress also caused a significant decrease in tiller production, TGW, BY, GY and harvest index (HI) (Table 1). Nevertheless, BC + ZnO-NPs mitigated the negative impacts of Sb on rice plants. Notably, BC + ZnO-NPs significantly enhanced tillers, TGW, BY, and GY by 17.14%, 32.56%, 34.68% and 22.54%, respectively, in Sb-polluted soil (Table 1).

**Table 1:** The effects of biochar and zinc nano-particles on morphological and yield traits of rice grown in Sb contaminated soil

Treatments	RL (cm)	RFW (g)	RDW (g)	PH (cm)	TPP	TGW (g)	BY/pot (g)	GY/pot (g)	HI (%)
Control	34.97 ± 1.25c	9.17 ± 0.08d	4.25 ± 0.14a	91 ± 3.30c	42 ± 0.82 cd	18.69 ± 0.40d	151.48 ± 1.74c	33.71 ± 1.29c	22.26 ± 1.08bcd
BC	46.23 ± 1.29b	12.69 ± 0.42b	5.22 ± 0.14ab	101 ± 1.25a	48 ± 1.63ab	23.69 ± 0.51b	174.57 ± 4.21ab	42.39 ± 0.83ab	24.29 ± 0.12ab
ZnO-NPs	42.60 ± 1.30b	11.17 ± 0.49c	4.91 ± 0.13b	98 ± 1.89ab	45 ± 1.70bc	21.20 ± 0.82c	166.52 ± 4.16b	38.87 ± 1.18b	23.38 ± 1.28abc
BC + ZnO-NPs	51.39 ± 1.98a	14.16 ± 0.55a	5.79 ± 0.12a	106 ± 2.49a	51 ± 0.82a	27.92 ± 0.36a	184.51 ± 3.71a	46.15 ± 1.25a	25.02 ± 0.71a
Sb	21.87 ± 1.69e	6.38 ± 0.21f	3.03 ± 0.08e	62 ± 2.45e	35 ± 0.82e	12.19 ± 0.21 g	113.96 ± 4.03f	22.26 ± 0.92f	19.54 ± 0.49e
Sb + BC	25.92 ± 1.26de	8.45 ± 0.25de	3.73 ± 0.42 cd	72 ± 2.05d	38 ± 2.45de	15.83 ± 0.51e	134.17 ± 3.74de	26.92 ± 1.28de	20.05 ± 0.39de
Sb + ZnO-NPs	22.23 ± 0.86e	7.51 ± 0.08ef	3.12 ± 0.61de	66 ± 2.49de	35 ± 1.25e	14.02 ± 0.56f	125.59 ± 2.97ef	24.35 ± 0.94ef	19.38 ± 0.30e
Sb + BC + ZnO-NPs	29.99 ± 1.08d	9.37 ± 0.19e	4.04 ± 0.083c	82 ± 2.16c	41 ± 1.64 cd	16.16 ± 0.39e	139.61 ± 2.95 cd	29.98 ± 1.21 cd	21.48 ± 0.78cde

The data given in columns is mean with three replications (± SD) and different letters indicating the significance among different treatments by Tukey test (*p < 0.05*)

*RL:* Root length, *RFW:* Root fresh weight, *RDW:* Root dry weight, *PH:* Plant height, *TPP:* Tillers/pot, *TGW:* 1000 grain weight, *BY:* biological yield, *GY:* Grain yield, *HI:* Harvest index

### Leaf photosynthetic pigments and water contents

**Fig. 2:**
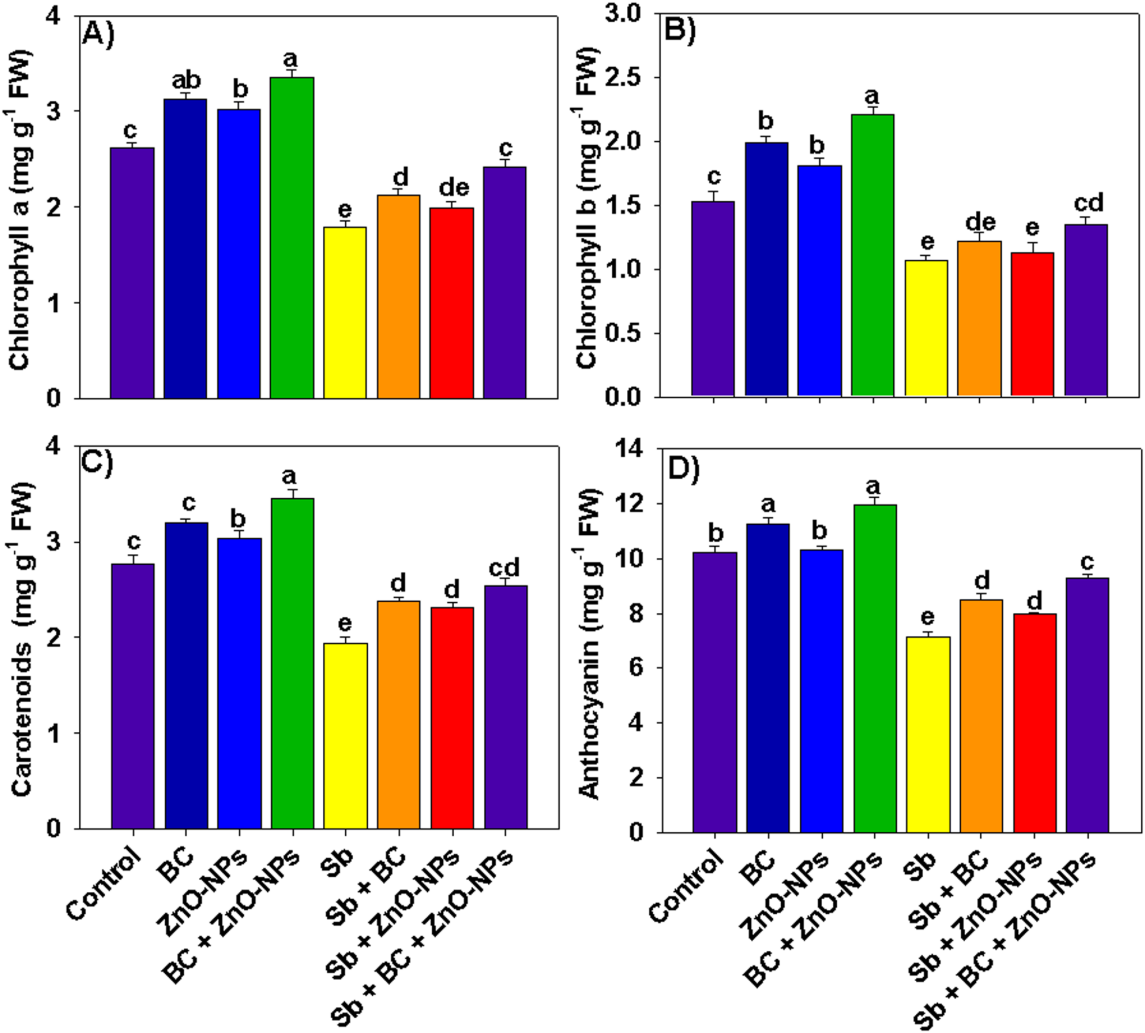
The effects of biochar and zinc nano-particles on chlorophyll (**a** and **b**), carotenoids (**c**) and anthocyanin (**d**) contents of rice grown in Sb contaminated soil. The data given in columns is mean with three replications (± SD) and different letters indicating the significance among different treatments by Tukey’s test (*p < 0.05*)

Biochar and ZnO-NPs significantly enhanced the leaf photosynthetic pigments synthesis under Sb stress (Fig. 2). Results depicted that BC + ZnO-NPs enhanced Chl-a, Chl-b, carotenoids, and anthocyanin synthesis by 68.44%, 94.60%, 49.35% and 50%, respectively, in Sb-contaminated soil (Fig. 2). Leaf RWC was significantly decreased in plants facing the Sb stress, while BC, ZnO-NPs, and their combination increased RWC by 7.02%, 3.64%, and 11.88%, respectively, in Sb-contaminated soil (Table 2).

**Table 2:** The effects of biochar and zinc nano-particles on leaf water contents, oxidative markers and osmolytes synthesis

Treatments	RWC (%)	EL (%)	MDA (µmol g⁻¹ FW)	H_2_O_2_ (µmol g⁻¹ FW)	TSP (mg g⁻¹ FW)	FAA (mg g⁻¹ FW)	Proline (mg g⁻¹ FW)
Control	72 ± 1.24bc	7.77 ± 0.48 cd	5.02 ± 0.16e	3.79 ± 0.108 g	11.48 ± 0.52d	12.40 ± 0.29d	1.03 ± 0.062cde
BC	78 ± 1.25a	9.30 ± 0.82 cd	5.34 ± 0.10e	4.12 ± 0.062ed	14.10 ± 0.27b	16.13 ± 0.35b	0.93 ± 0.042def
ZnO-NPs	76 ± 0.94ab	11.22 ± 0.81c	6.16 ± 0.08d	4.37 ± 0.156e	12.78 ± 0.52c	13.99 ± 0.42c	0.82 ± 0.029f
BC + ZnO-NPs	81 ± 2.49a	6.26 ± 2.36d	4.17 ± 0.12f	3.35 ± 0.109 fg	15.75 ± 0.57a	17.40 ± 0.60a	0.73 ± 0.022ef
Sb	62 ± 1.70e	51.63 ± 1.15a	8.96 ± 0.22a	9.19 ± 0.233a	7.25 ± 0.11 g	7.27 ± 0.16 g	1.09 ± 0.074 cd
Sb + BC	66 ± 1.88cde	44.00 ± 2.09b	8.08 ± 0.08bc	8.02 ± 0.100c	9.34 ± 0.24ef	9.34 ± 0.16f	1.35 ± 0.050ab
Sb + ZnO-NPs	64 ± 1.63de	49.16 ± 1.28a	8.43 ± 0.18b	8.53 ± 0.090b	8.67 ± 0.23f	8.40 ± 0.21 fg	1.21 ± 0.060bc
Sb + BC + ZnO-NPs	69 ± 2.16 cd	41.08 ± 1.24b	7.76 ± 0.18c	7.29 ± 0.011d	10.39 ± 0.12de	10.66 ± 0.21e	1.58 ± 0.0130a

The data given in columns is mean with three replications (± SD) and different letters indicating the significance among different treatments by Tukey test (*p < 0.05*)

*RWC:* Relative water contents, *EL:* Electrolyte leakage, *MDA:* Malondialdehyde, *H*_2_O_2_: Hydrogen peroxide, *TSA:* Total soluble proteins, *FAA:* Free amino acids

### Oxidative stress markers, and osmolytes

The maximum concentration of H_2_O_2_ (9.19 µ mol g^− 1^), MDA (8.96 µ mol g^− 1^) and EL (51.63%) was observed in Sb treatment receiving no BC and ZnO-NPs application (Table 2). Plant receiving the BC + ZnO-NPs depicted a significant decrease in H_2_O_2_ (26.06%) MDA (15.46%), and EL (25.68%) production (Table 2). Antimony inhibited TSP and FAA synthesis; in contrast, it augmented proline synthesis (Table 2). Plants raised with BC + ZnO-NPs showed maximum TSP, FAA and proline contents under Sb stress (Table 2).

### Hormones and antioxidants

**Fig. 3:**
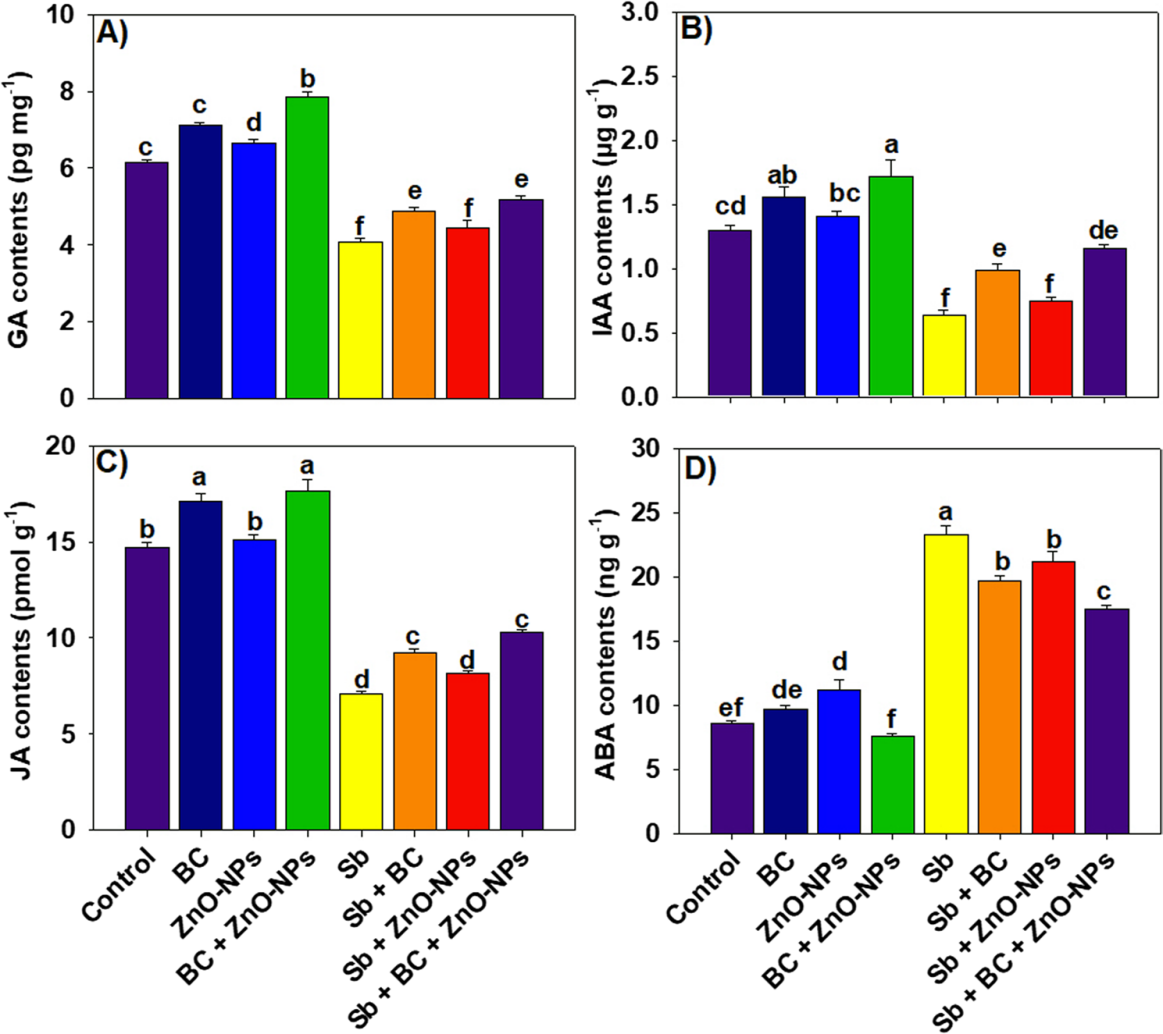
The effects of biochar and zinc nano-particles on GA (**a**), IAA (**b**), JS (**c**), and ABA (**d**) synthesis in rice grown in Sb contaminated soil. The data given in columns is mean with three replications (± SD) and different letters indicating the significance among different treatments by Tukey’s test (*p < 0.05*). GA: gibberellin, IAA: indole acetic acid, JS: jasmonic acid and ABA: abscisic acid

**Fig. 4:**
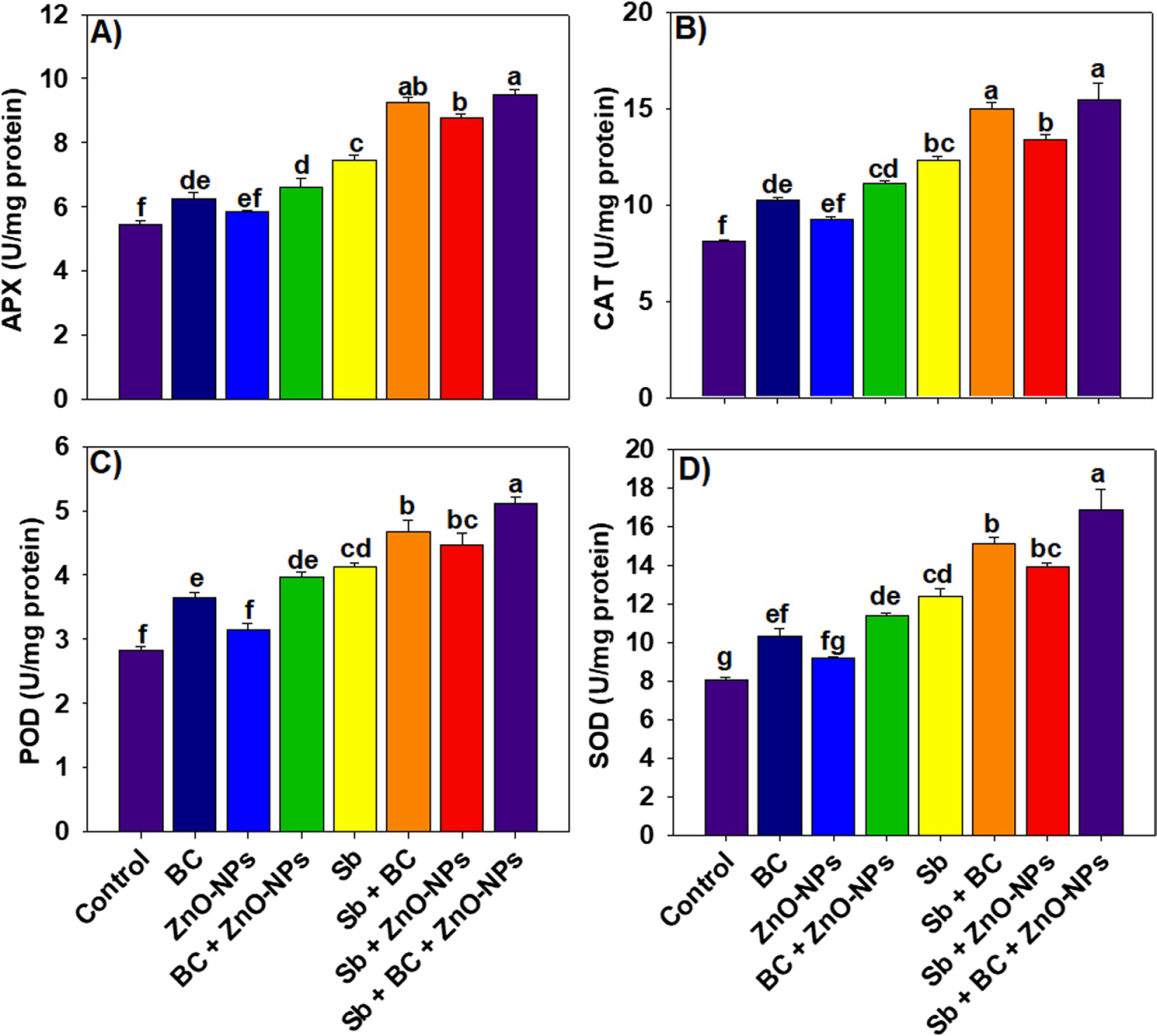
The effects of biochar and zinc nano-particles on APX (**a**), CAT (**b**), POD (**c**), and SOD (**d**) activities of rice grown in Sb contaminated soil. The data given in columns is mean with three replications (± SD) and different letters indicating the significance among different treatments by Tukey’s test (*p < 0.05*). APX: ascorbate peroxide, CAT: catalase, POD: peroxidase, SOD: super oxide dismutase

The synthesis of GA, IAA, and JA was significantly decreased by 51.10%, 103% and 96.81%, however, BC + ZnO-NPs enhanced synthesis of these hormones (Fig. 3). Abscisic acid showed a different trend, and its concentration was increased under Sb stress, while BC (18.25%), ZnO-NPs (9.61%) and their co-application (32.53%) decreased ABA synthesis (Fig. 3). Overall, the lowest APX, CAT, POD, and SOD activity was observed in control, while antioxidant activity was increased in response to Sb. Notably, BC + ZnO-NPs improved APX, CAT, POD, and SOD activities by 27.38% 25.87%, 23.78%, and 35.66%, respectively (Fig. 4).

### Tissue nutrients and antimony accumulation in plant tissues

Antimony stress decreased the accumulation of N, P, and K in rice seedlings by 52.13%, 60.02% and 83.22%, respectively (Table S2). All the treatments, particularly co-applied BC + ZnO-NPs, enhanced nutrients accretion under Sb stress (Table S2). Results showed that Sb accumulation was increased in plants receiving no BC + ZnO-NPs application (Table S2). Both BC + ZnO-NPs significantly diminished Sb concentration in root and shoot by 48.12% and 65.73% respectively, (Table S2).

### Gene expression and iron plaque formation

**Fig. 5:**
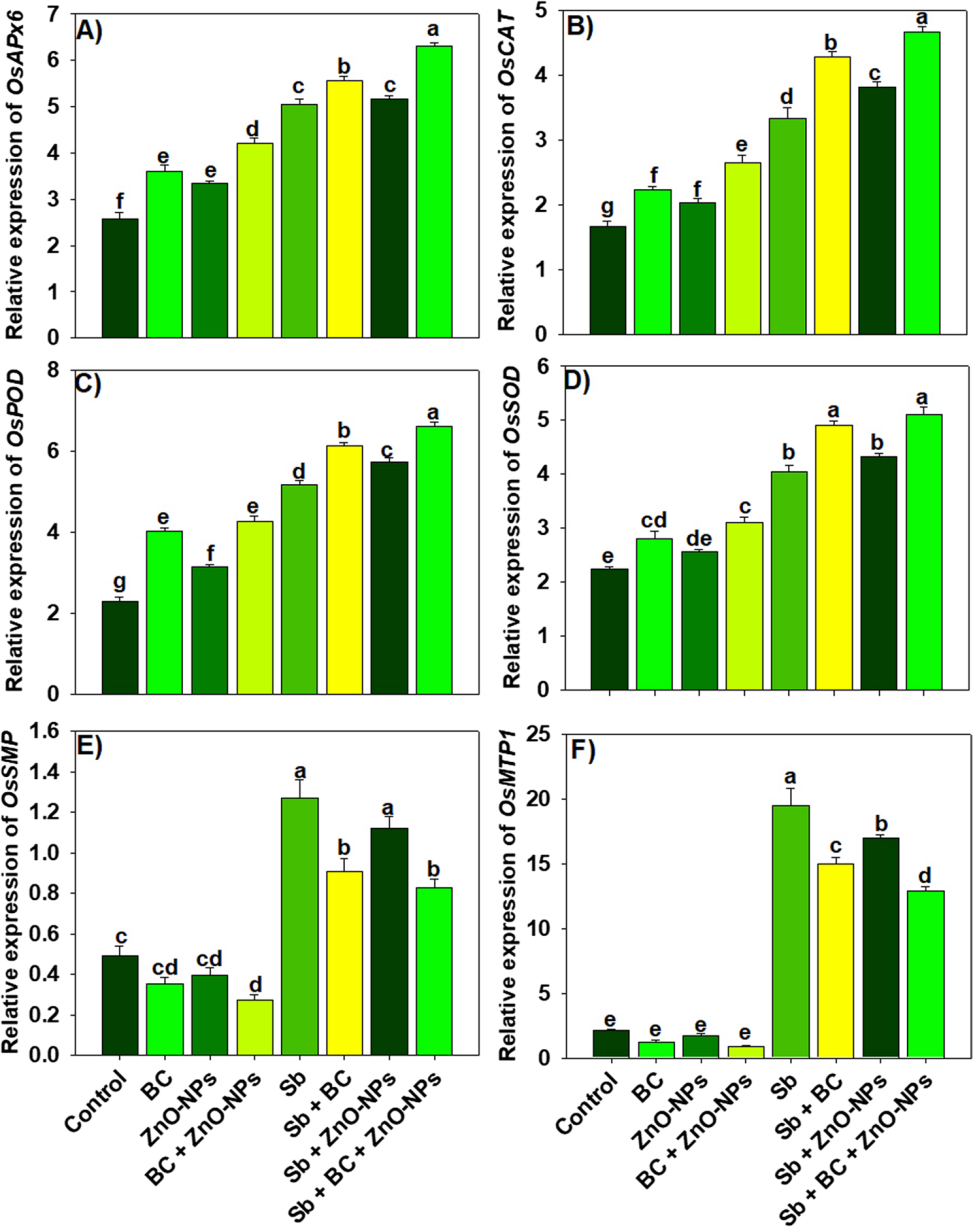
The effects of biochar and zinc nano-particles on antioxidants gene expression (**a**-**d**) and Sb uptake genes (**e**-**f**) of rice grown in Sb contaminated soil. The data given in columns is mean with three replications (± SD) and different letters indicating the significance among different treatments by Tukey’s test (*p < 0.05*)

**Fig. 6:**
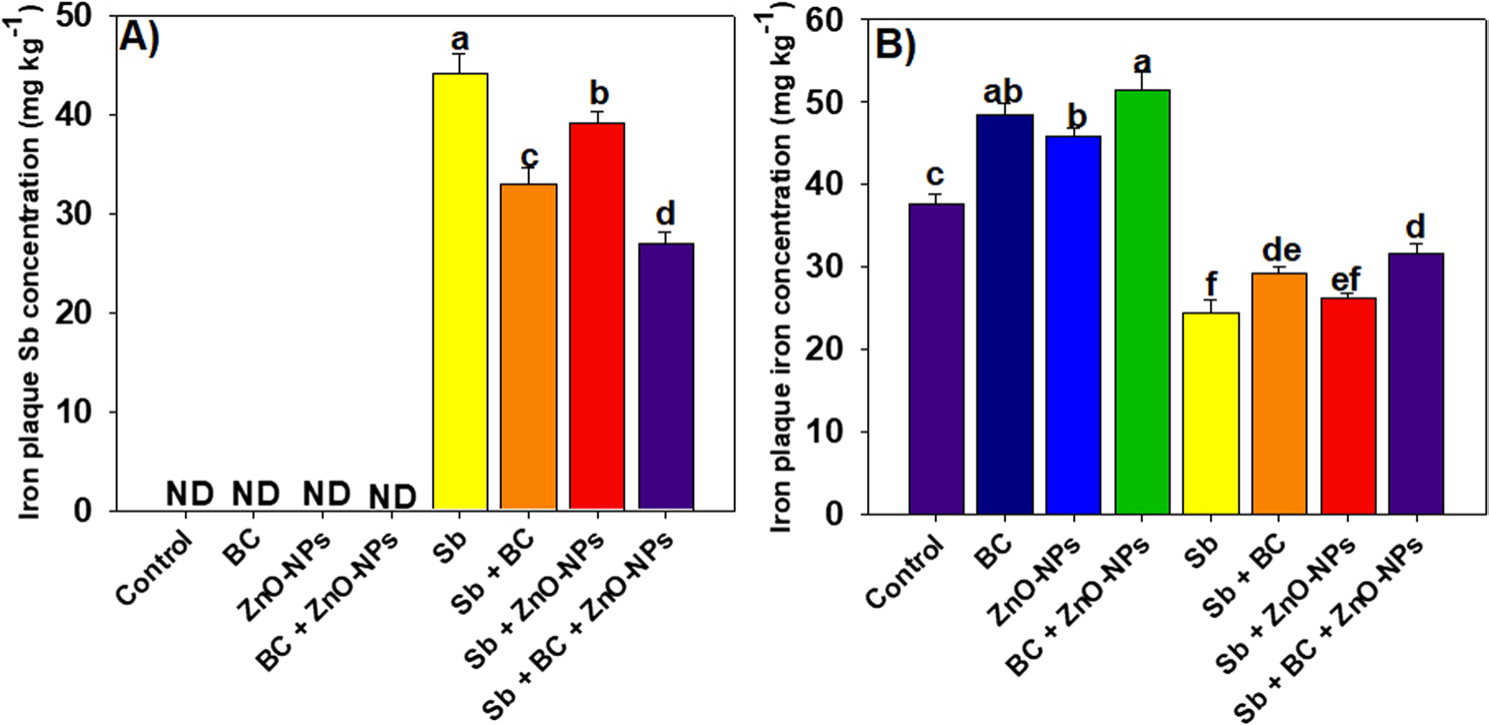
The effects of biochar and zinc nano-particles on Sb (**a**) and Fe (**b**) concentration in roots ACA extract. The data given in columns is mean with three replications (± SD) and different letters indicating the significance among different treatments by Tukey’s test (*p < 0.05*)

Antioxidant gene expression was increased due to Sb stress BC and ZnO-NPs application (Fig. 5). Notably, BC + ZnO-NPs enhanced the *OsAPx6*, *OsCAT*, *OsPOD*, and *OsSOD* expression level by 24.95% 39.93%, 27.60% and 25.92%, respectively, under Sb stress (Fig. 5). BC + ZnO-NPs also reduced the expression of Sb uptake genes (*OsSMP* and *OsMTP1*) by 53.01% and 51.35% respectively, in plants facing Sb stress (Fig. 5). The results of the ascorbic citrate acetic (ACA) extract analysis showed BC + ZnO-NPs decreased the iron plaque Sb and increased iron plaque Fe concentration (Fig. 6). Notably, BC + ZnO-NPs decreased Sb concentration by 63.60% in ACA extract, while it increased the Fe concentration by 29.87% in ACA extract (Fig. 6).

### Soil antimony, nutrient availability and enzymes activity

Biochar, ZnO-NPs, and their integration decreased soil Sb availability by 23.45%, 9.93%, and 41.80%, respectively (Table S3). Antimony decreased the soil pH; however, BC and ZnO-NPs supply increased the soil pH (Table S3). Antimony stress also markedly reduced TN, AP, and AK availability by 63.45%, 71.51%, and 38.99%, respectively (Table S3). Biochar and ZnO-NPs significantly enhanced nutrient availability; the best results were observed with combined BC + ZnO-NPs application (Table S3). Antimony stress negatively affected the soil enzyme activity and decreased urease and catalase activities by 108.35% and 50%, respectively (Table S2). The results showed that the treatment with BC + ZnO-NPs showed the greatest increase of 73.37% and 40.74% in soil urease and catalase activities under Sb stress (Table S3).

## Discussion

Antimony caused a marked reduction in rice yield, which was linked to impaired plant functioning, increased Sb accumulation, and inhibited nutrient uptake (Table S1), aligning with previous studies by Daji et al. [48]. Since Sb is non-essential for plants, its accumulation induced excessive H₂O₂ production and membrane damage, as evidenced by increased MDA and EL thereby causing yield losses through the loss of essential solutes [31, 48, 49]. Earlier findings also revealed that Sb accumulation induces oxidative stress and membrane damage, leading to poor growth [50]. In addition, Sb decreases soil nutrient availability and accumulated in plant tissues, which disrupts plant functioning and results in yield losses [8]. BC + ZnO-NPs significantly enhanced rice biomass and yield (Table 1). This synergistic effect was mediated through a series of mechanisms. Firstly, BC + ZnO-NPs decreased oxidative stress (Table 2), which enhanced chlorophyll contents [51], resulting in improved photosynthetic efficiency and plant growth. Additionally, the combined treatment improved leaf water content and restored hormonal balance by decreasing ABA synthesis while increasing GA, IAA, and JA synthesis, collectively promoting better growth [8]. Secondly, BC + ZnO-NPs decreased lipid peroxidation by reducing MDA production through enhanced antioxidant activities and limited Sb uptake, thereby protecting membrane integrity [52]. This membrane-stabilizing effect may have ensured better cell survival under Sb stress and contributed to improved growth. Thirdly, BC + ZnO-NPs upregulated antioxidant enzymes, which helped in counteracting Sb toxicity [53]. Thus, this coordinated antioxidant response enabled rice plants to counteract Sb-induced oxidative stress more effectively than individual treatments.

Chlorophyll plays a crucial role in photosynthesis, carbon assimilation assimilation, and subsequent growth [8]. In this study, Sb decreased chlorophyll synthesis (Fig. 2) by increasing oxidative damage. Antimony targets the SH groups of chloroplast proteins, which undermines chloroplast stability and impairs enzymes activity linked to chlorophyll synthesis [11]. Antimony also reduces photosystem II (PS-II) efficiency and alters thylakoid membrane fluidity by increasing ROS production, collectively diminishing photosynthetic efficiency and leading to reduced growth and yield [48]. Our findings demonstrate that the BC + ZnO-NPs alleviated these inhibitory effects. This treatment enhanced magnesium uptake, which supports chlorophyll synthesis [51], while simultaneously reducing oxidative stress. This resulted in improved photosynthetic efficiency and overall plant growth under Sb stress.

The activities of antioxidants were increased under Sb stress, suggesting the activation of antioxidant defense mechanisms to counteract Sb toxicity. We observed that BC + ZnO-NPs synergistically enhanced antioxidants through complementary mechanisms. Biochar immobilized Sb and reduced its uptake and subsequent ROS production, whereas ZnO-NPs may have supplied zinc, which is a cofactor for antioxidant enzymes. Therefore, improved soil environment, reduced Sb availability, and enhanced zinc availability contributed to the increase in antioxidant activity. Proline aids in maintaining osmotic balance, and in the current study, BC + ZnO-NPs enhanced proline synthesis under Sb stress (Table 2). This increase in proline synthesis indicates a protective response employed by rice plants against the Sb-mediated decrease in solute potential. Proline works as an osmolyte and ROS scavenger, stabilizing cellular membranes and proteins under stress conditions. Therefore, its increased accumulation after BC + ZnO-NPs application indicates an adaptive stress response, improving Sb tolerance in rice plants. Antimony decreased TSP and FAA through interconnected mechanisms, including increased ROS production, which causes protein oxidation and fragmentation [48]. Simultaneously, Sb disturbed nitrogen metabolism and reduced nitrogen availability, which is essential for protein and amino acid synthesis. BC + ZnO-NPs enhanced the synthesis of SP and FAA by decreasing Sb availability, which mitigated ROS and may have reduced protein degradation and oxidation. This combined treatment also enhanced soil nitrogen availability, which plays a crucial role in the synthesis of both FAA and TSP. It is also plausible that the BC + ZnO-NPs-mediated decrease in ABA production restored hormonal balance and promoted metabolic activity favoring amino acid biosynthesis.

The increased synthesis of osmolytes (FAA and TSP) scavenges the ROS and diminishes the membrane damage, thereby leading to an increase in growth [54, 55]. Hormones play a crucial role in plant growth and stress tolerance. The synthesis of GA, IAA, and JA was significantly enhanced after BC + ZnO-NPs application (Fig. 3). This increase suggests that BC + ZnO-NPs may have activated regulatory signaling pathway genes involved in hormone biosynthesis, thereby promoting the production of these growth-promoting hormones [56, 57]. Hormones such as IAA aid in root growth. The BC + ZnO-NPs treatment elevated IAA levels, which enhanced root growth, improved water and nutrient absorption, and ultimately supported overall plant growth. Conversely, Sb elevated ABA synthesis, which is typically linked to stomatal closure and growth inhibition. Our findings showed that BC + ZnO-NPs effectively reduced ABA synthesis, which likely promoted stomatal opening and sustained better photosynthetic efficiency and overall plant growth.

Biochar + ZnO-NPs significantly decreased Sb accumulation in plant tissues. Biochar has a larger surface and functional groups (Fig. 1), and its application might have caused the adsorption of Sb, thus reducing its availability and accumulation [31]. Biochar also increases soil pH, which immobilizes Sb and subsequently decreases its accumulation in plant organs [58]. Additionally, BC facilitates ionic exchange with root cytoplasm, mediated by specific binders [59], which in turn reduces metal accumulation in plant tissues. Nanoparticles, due to their small size, freely access cell walls and penetrate seeds and roots [60]. Consequently, NPs facilitate the chelation of toxic metals, thereby decreases their accretion. The increase in Zn²⁺ availability from from NPs may also decreased the uptake of Sb by competing it with at exchange sites. This interaction indicates the synergistic impacts BC + ZnO-NPs in reducing Sb uptake, and its subsequent accumulation in plant tissues. Biochar + ZnO-NPs significantly decreased the expression of Sb uptake genes, leading to decrease in Sb uptake and accumulation. The impacts of BC + ZnO-NPs on gene expression could be varied according to stress treatment and duration, and the genetic background of the crop varieties. Therefore, studies are required to comprehend how BC + ZnO-NPs affects the gene expression involved in Sb uptake.

Antimony decreased the soil pH, while BC + ZnO-NPs synergistically enhanced the soil pH by releasing the alkaline compounds present in BC [61, 62]. Antimony significantly decreased the soil nutrient availability (Table S3), possibly by decreasing soil microbial and enzyme activity involved in nitrogen cycling [4]. In contrast, BC + ZnO-NPs significantly enhanced the soil nutrient availability. This increase was linked with BC + ZnO-NPs mediated improvement in soil structure and carbon availability [63]. We also observed that BC and ZnO-NPs acted synergistically to reduce the availability of Sb in the soil. This reduction lessened the competition between Sb and essential nutrients, thereby increasing nutrient availability [31, 64]. Biochar used in the current study contained an appreciable amount of nutrients, thus its application also directly enhanced the soil nutrient availability. Antimony stress decreased the soil enzyme activity (Table S3), due to inhibited growth of bacteria and fungi involved in enzyme synthesis [65]. Antimony toxicity alters the enzyme’s structure and stability by increasing ROS production and inhibiting microbial activities [66, 67]. This study showed BC + ZnO-NPs improved urease and catalase activity by decreasing the oxidative damage and increasing the soil nutrient availability. This indicates a complex interaction between BC + ZnO-NPs and soil nutrient cycling. Biochar + ZnO-NPs synergistically decreased the soil Sb availability. This was achieved through an increase in soil pH, which causes Sb immobilization, thus decreasing its availability. Biochar application also enhanced SOC which fix Sb in soil, thus decreases its availability [31]. Besides this, NPs also cause chelation and immobilization of toxic metals, thus reducing their availability [68]. Therefore, synergistic application of BC + ZnO-NPs achieved better results in decreasing the soil Sb availability. The Sb concentration (250 mg kg⁻¹) used in the current study was selected based on Sb contamination levels near mining areas and their surrounding agricultural soils. For instance, in the Xikuangshan mine of Hunan Province, soil Sb concentrations reached 248–1584 mg kg⁻¹, which are significantly higher than the maximum allowable levels [6, 69]. Therefore, the selected Sb concentration (250 mg kg⁻¹) represents a realistic worst-case scenario commonly encountered in agricultural soils near mining and industrial areas. This makes the current finding environmentally relevant and applicable to regions facing Sb contamination.

## Conclusion

This study demonstrates that antimony inhibited rice growth and productivity by inducing oxidative stress, causing membrane damage, disrupting hormonal balance, and promoting antimony accumulation in plant tissues. In contrast, biochar and zinc nano-particles effectively counteracted antimony-induced toxicity. This partnership enhanced rice productivity by increasing chlorophyll synthesis and antioxidant activity, restoring hormonal balance, and promoting iron plaque formation, which reduced antimony accumulation in plant tissues. Collectively, these findings establish that the biochar–nanoparticle combination is a promising strategy to mitigate antimony toxicity. This approach is consistent with the wider objectives of sustainable farming by offering dual benefits: remediating contaminated soils while increasing crop productivity. However, future field trials are necessary to validate these results before making practical recommendations. Moreover, molecular studies are needed to fully understand the mechanisms through which the combined use of biochar and nanoparticles alleviates antimony stress.

## Supplementary Information

Supplementary Material 1.

## Data Availability

The data is contained within the article. Further correspondence can be made to the corresponding author.
