# Supplementary material for: Biochar and zinc oxide nanoparticles partnership: a multi-faceted strategy to enhance rice productivity and remediate antimony polluted soils
Source: BMC Plant Biol. 2026 Jun 12;26:1413. doi: 10.1186/s12870-026-09069-6 (PMC13483681; doi:10.1186/s12870-026-09069-6)
Supplement: Supplementary file 1 [file 12870_2026_9069_MOESM1_ESM.docx]

**Table S1: Primers used in gene expression analysis**

| **Gene** | **Details of primers** |
| --- | --- |
| OSPOX-F | GTTGCCTGTTGATGCTCTGCT |
| OsPOX-R | CCGCCTGTGCTACGATGG |
| OsCAT-F | CAACCACTACGACGGCTTCA |
| OsCAT-R | TCCTTGGCAATCACCACCTT |
| OsAPX-F | GGATGGGGCAAACCAGAAA |
| OsAPX-R | GGTCCTCATCTCGGCGTTC |
| OsSOD-F | GCTGCCAGTTTGACTACAAATACC |
| OsSOD-R | AATGTGATCTCAGGCGACCC |
| OsMTP1-F | CCTGGTTCGGTTAGGGTGG |
| OsMTP1-R | CTGCATTAGCCGCATGTTTTA |
| OsSMP-F | CCGCAACGACCCCAACTA |
| OsSMP-R | CTGGCGACACCATCCCTG |
| Actin-F | CATTGGTGCTGAGCGTTTCC |
| Actin-R | CCCGCAGCTTCCATTCCTAT |

**Table S2:** The effects of biochar and zinc nano-particles on concentration of nitrogen, phosphorus and potassium in rice seedlings grown in Sb polluted soil

| **Treatments** | **Nitrogen**  **(mg kg^-1^ DW)** | **Phosphorous**  **(mg kg^-1^ DW)** | **Potassium**  **(mg kg^-1^ DW)** | **Root-Sb (mg kg^-1^)** | **Shoot-Sb (mg kg^-1^)** |
| --- | --- | --- | --- | --- | --- |
| Control | 23.55±1.26cd | 15.17±0.19cd | 32.98±1.69cd | ND | ND |
| BC | 32.93±1.73ab | 18.22±0.82b | 38.51±1.50b | ND | ND |
| ZnO-NPs | 27.85±1.20bc | 16.10±0.32bc | 36.40±0.82bc | ND | ND |
| BC + ZnO-NPs | 35.99±3.30a | 21.95±1.63a | 43.11±1.70a | ND | ND |
| Sb | 15.48±0.69f | 9.48±0.45f | 18.00±1.10g | 33.98±1.23a | 19.22±0.82a |
| Sb + BC | 19.22±0.82def | 12.36±0.099e | 24.02±1.34ef | 25.39±0.73c | 13.30±0.74c |
| Sb + ZnO-NPs | 17.18±0.77ef | 10.85±0.45ef | 21.22±0.82fg | 29.22±0.81b | 15.47±0.86b |
| Sb + BC + ZnO-NPs | 21.21±0.81de | 13.19±0.12de | 28.52±1.20de | 22.94±1.78c | 11.81±0.31c |

The data given in columns is mean with three replications (±SD) and different letters indicating the significance among different treatments by Tukey HSD test (*p < 0.05*).

**Table S3:** The effects of biochar and zinc nano-particles on Sb accumulation in plant organs, soil Sb and nutrients availability and soil enzymes activity after harvesting rice crop

| **Treatments** | Soil-Sb (mg kg^-1^) | Soil pH | Soil TN (g kg^-1^) | Soil AP (g kg^-1^) | Soil AP (g kg^-1^) | **Urease (mg NH_4_^+^ -N g^−1^ day^−1^)** | **Catalase (1 µ mol H_2_O_2_ g^−1^ day^−1^)** |
| --- | --- | --- | --- | --- | --- | --- | --- |
| Control | ND | 5.78±0.043bc | 1.43±0.025c | 26.55±0.80bc | 107.01±2.34bc | 0.67±0.045cd | 13.29±0.82c |
| BC | ND | 5.96±0.036ab | 1.74±0.043ab | 32.97±1.25a | 119.22±2.16ab | 0.86±0.033ab | 17.66±0.42ab |
| ZnO-NPs | ND | 5.86±0.029ab | 1.61±0.033b | 28.76±1.12b | 116.16±3.06ab | 0.76±0.045bc | 15.90±0.50b |
| BC + ZnO-NPs | ND | 6.02±0.096a | 1.89±0.045a | 35.38±1.62a | 129.24±6.51a | 0.95±0.043a | 18.58±0.94a |
| Sb | 90.33±1.99a | 5.36±0.062e | 0.87±0.041d | 15.48±0.32f | 77.01±3.44e | 0.32±0.025f | 8.86±0.19f |
| Sb + BC | 73.17±2.05c | 5.49±0.033de | 1.06±0.033de | 19.55±1.24de | 87.08±4.05de | 0.48±0.029e | 10.73±0.33de |
| Sb + ZnO-NPs | 82.17±2.56b | 5.43±0.060de | 0.92±0.057ef | 17.79±1.11ef | 83.22±3.56e | 0.43±0.021ef | 9.89±0.27ef |
| Sb + BC + ZnO-NPs | 63.70±1.37d | 5.62±0.053cd | 1.15±0.073d | 22.60±1.34cd | 98.51±4.47cd | 0.56±0.046de | 12.47±0.23cd |

The data given in columns is mean with three replications (±SD) and different letters indicating the significance among different treatments by Tukey HSD test (*p < 0.05*). TN: total nitrogen, AP: available phosphorous, AK: available potassium.
